# Association Between Visual Search Performance and Eye–Hand Coordination in School-Aged Children

**DOI:** 10.3390/life16091421

**Published:** 2026-08-27

**Authors:** Daisuke Sudo

**Affiliations:** National Institute of Advanced Industrial Science and Technology (AIST), Kashiwa II Campus, University of Tokyo, Chiba 277-0882, Japan; daisuke-sudou@aist.go.jp

**Keywords:** eye–hand coordination, trail making test, ruler drop test, visual search, visuomotor development

## Abstract

Eye–hand coordination (EHC), a fundamental component of visuomotor development in children, plays an important role in motor control, learning, and injury prevention. This study aimed to examine the association between trail making test part A (TMT-A) performance and EHC in school-aged children. Twenty-seven children aged 9–14 years participated in this cross-sectional study. EHC was assessed using a touchscreen-based visuomotor task, whereas visual search and reaction speed were evaluated using the TMT-A and ruler drop test (RDT), respectively. Spearman’s correlation and multiple linear regression analyses were performed with adjustment for age and sex. Bias-corrected and accelerated (BCa) bootstrap confidence intervals (2000 resamples) were calculated. Both TMT-A and RDT were significantly correlated with EHC performance. In the full sample, TMT-A remained significantly associated with EHC performance after adjustment for age and sex; however, this association was not robust in a sensitivity analysis excluding a high-leverage observation. Thus, the adjusted association should be considered exploratory and requires confirmation in larger independent samples.

## 1. Introduction

Eye–hand coordination (EHC) is an important component of visuomotor development in children that contributes to motor control, learning, and everyday goal-directed actions. Successful EHC requires the integration of visual information with timely and accurate upper-limb responses. Understanding the cognitive and visuomotor processes associated with EHC may therefore contribute to the characterization of developmental performance in school-aged children.

In developmental assessments, visual function is often evaluated using only visual acuity tests. However, the importance of comprehensive visual function is being increasingly recognized. In particular, elements, such as eye–hand coordination (EHC), visual exploration, and attention control, are intricately involved not only in learning and daily life but also in motor control, balance maintenance, and injury prevention [1,2,3]. EHC refers to the ability to integrate visual information with goal-directed hand movements for rapidly and accurately detecting and responding to visual targets [4]. Operationally, EHC can be assessed using a touchscreen-based visuomotor task requiring participants to visually identify and manually respond to randomly presented targets.

EHC is closely associated with cognitive abilities across developmental stages. For example, Chan et al. demonstrated that interactive cognitive–motor training improved both EHC and cognitive performance [5,6]. EHC is a core component of visuomotor control that contributes to both cognitive function and motor regulation, which make it an essential skill in child development [4,7].

Established pediatric assessments, such as the Beery–Buktenica Developmental Test of Visual–Motor Integration, Sixth Edition (Beery VMI), provide standardized measures of visual–motor integration and include supplementary assessments of visual perception and motor coordination [8].

Pediatric research indicates that trail making test (TMT) performance changes substantially with the age of individuals, including Japanese children and adolescents [9]. However, pediatric studies have differed in stimulus number, layout, and administration procedures. The Japanese developmental study used a simplified form with fewer stimuli than the conventional TMT [9], and research in typically developing youth aged 9–14 years used the Halstead–Reitan intermediate form rather than a version identical to the conventional test administered to adults [10]. Moreover, the standardized Japanese TMT-J provides normative interpretation only for individuals aged 20–89 years [11].

EHC develops rapidly from early childhood through adolescence and is regarded a foundational component of cognitive and motor competence in children [1,2]. Various methods have been used to assess EHC. The traditional TMT evaluates visual search, attention shifting, and sequencing and has been widely employed as an index of visual exploration and attention control [7,9,10]. Similarly, the ruler drop test (RDT) is a simple and practical measure used to assess reaction time in response to visual stimuli [12,13]. Taken together, previous studies have indicated that EHC is a critical factor in understanding development and motor learning in children. Although several visuomotor assessment tools are available, many require specialized materials, equipment, or examiner training. Therefore, simple and practical methods that can be readily implemented in community and educational settings are desirable. Although visual functions, such as visual exploration and EHC, play important roles in daily life and learning, practical assessment approaches that can be easily applied outside specialized clinical settings are warranted.

The TMT-A is often described as a visual search task because it requires participants to locate and connect sequentially numbered targets. However, performance on this test reflects multiple cognitive and motor components, including processing speed, sustained attention, visuospatial ability, sequencing, graphomotor speed, and executive functioning. These processes may be relevant to EHC because successful EHC also requires rapid detection of visual targets, allocation of attention, transformation of visual information into motor commands, and continuous adjustment of hand movements. Therefore, TMT-A is not a pure measure of visual search, but rather as a simple task that may capture broader cognitive–visuomotor processes associated with EHC.

This study aimed to examine the association between TMT-A performance and performance on a touchscreen-based visuomotor task in school-aged children. It was hypothesized that TMT-A completion time would be associated with touchscreen task completion time after adjustment for age and sex. The RDT was included as a measure of simple visuomotor reaction speed for exploratory comparison.

## 2. Materials and Methods

A total of 27 children (18 boys and 9 girls), aged 9–14 years, participated in this study. The participants were recruited between May and July 2025 through the Chiba Children’s University program organized by Sawayaka Chiba Citizen’s Plaza and were assessed at the National Institute of Advanced Industrial Science and Technology (AIST), Kashiwa Center, Japan, in August 2025. Convenience sampling was used. The program had a maximum capacity of 30 participants; 27 participants attended it and completed the study procedures. No financial or material incentives were provided.

The inclusion criteria required participants to have visual function sufficient for daily activities and no history of orthopedic conditions that could affect walking or motor performance. No objective visual acuity or ophthalmologic examination was performed at the study site. Eligibility with respect to visual function was determined based on confirmation from both the child and the child’s parent or guardian regarding the child having visual function sufficient for daily activities. The protocol for data collection was approved by the Ethics Committee of AIST (Approval No. 73012040-E-20250708-002).

### 2.1. Sample Size Justification

The sample size was modest (*n* = 27), and all regression analyses were, therefore, considered exploratory. Jenkins and Quintana-Ascencio suggested that approximately 25 observations may be sufficient under certain simulation conditions to reduce model-pattern misidentification; however, they did not eliminate concerns regarding statistical power, coefficient stability, model overfitting, or generalizability [14]. Therefore, the regression estimates were interpreted cautiously. Bootstrap confidence intervals (CIs) were used to provide a distribution-based description of uncertainty around the coefficient estimates. However, it should be acknowledged that this approach does not address limitations related to statistical power, sampling variability, or generalizability.

### 2.2. Informed Consent

Written informed consent was obtained from the parents of all the participants before initiating the study. The children were individually provided with age-appropriate explanations of the study procedures using simple language and explanatory materials, and their assent was obtained after confirming their understanding. The experimental procedures were explained both verbally and in writing, and written informed consent was obtained from each participant and the parents.

### 2.3. Experimental Procedures

Participants were divided into three groups and rotated through three visual–motor assessment stations on the same floor using the sequences ABC, BCA, and CAB. The touchscreen-based visuomotor task was administered at one station, whereas TMT-A and RDT were administered at another station. The third station involved ancillary physical-function assessments. Although this rotation avoided any single fixed order of stations, potential fatigue or carryover effects were not formally evaluated. The total assessment time was approximately 30 min, and formal rest periods were not scheduled during testing.

### 2.4. Visual Function Assessments

#### 2.4.1. EHC

EHC was assessed using the V-training system Ver2 (Advance Vision Partners LLC, Tokyo, Japan). The V-training system has been used in studies evaluating visuomotor and visual functions, including EHC and spatial awareness, in both younger and older populations [15,16].

**Figure 1:**
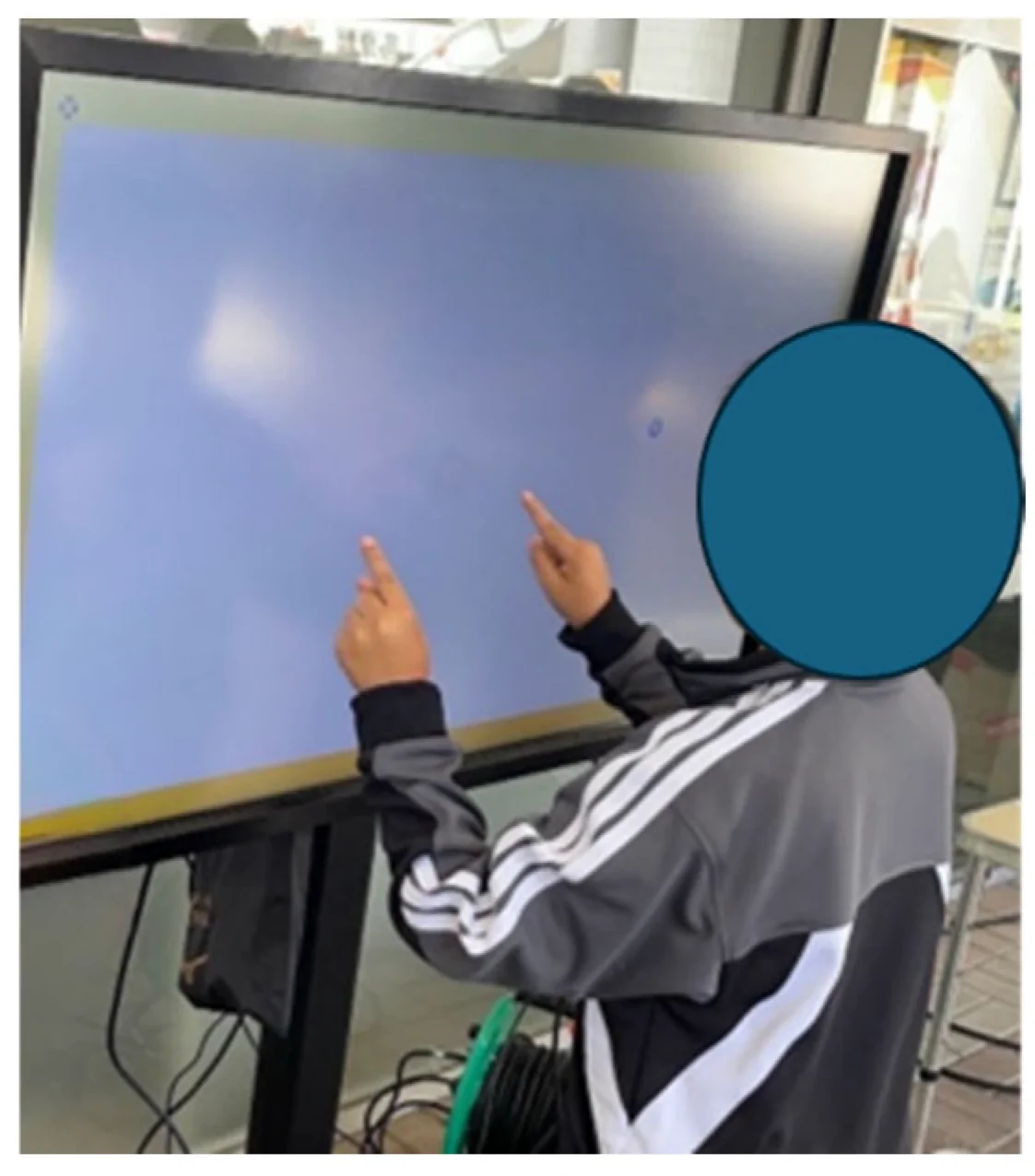
Eye–hand coordination task displayed on the touchscreen monitor (V-training system).

The participants stood approximately 40 cm from a 50-inch touchscreen monitor and visually tracked and touched 30 circular targets presented sequentially at random positions across the central and peripheral visual fields. Each target disappeared when touched, after which the next target appeared. The target locations were randomized and were not identical for the participants. The participants were allowed to use either hand and completed one practice trial before testing. The task consisted of a single test session in which participants attempted to eliminate all 30 targets as quickly as possible. The total completion time automatically generated by the V-training system was used as the predefined outcome measure (Figure 1). Trial-level performance, accuracy-related measures, and error data were not available for analysis.

#### 2.4.2. TMT-A

The TMT Part A (TMT-A) is a simple visual search and processing speed task in which participants are required to connect numbers from 1 to 25 in ascending order, as quickly as possible. This task reflects multiple cognitive and motor components, including visual search, processing speed, sustained attention, sequencing, visuospatial ability, graphomotor speed, and aspects of executive functioning.

Part A of the official Trail Making Test, Japanese Version (TMT-J; Shinko Igaku Shuppansha, Tokyo, Japan, 2019) was used [11]. TMT-A was selected instead of TMT-B because the study focused on number-sequencing performance and its association with the touchscreen task. The test was administered and scored in accordance with the standardized TMT-J procedures without adaptation or modification. Completion time was recorded as a continuous performance measure. Because the available TMT-J normative data apply only to adults, the results were not interpreted using normative cutoffs.

#### 2.4.3. RDT

The RDT is a simple method used to evaluate the reaction time to visual stimuli.

**Figure 2:**
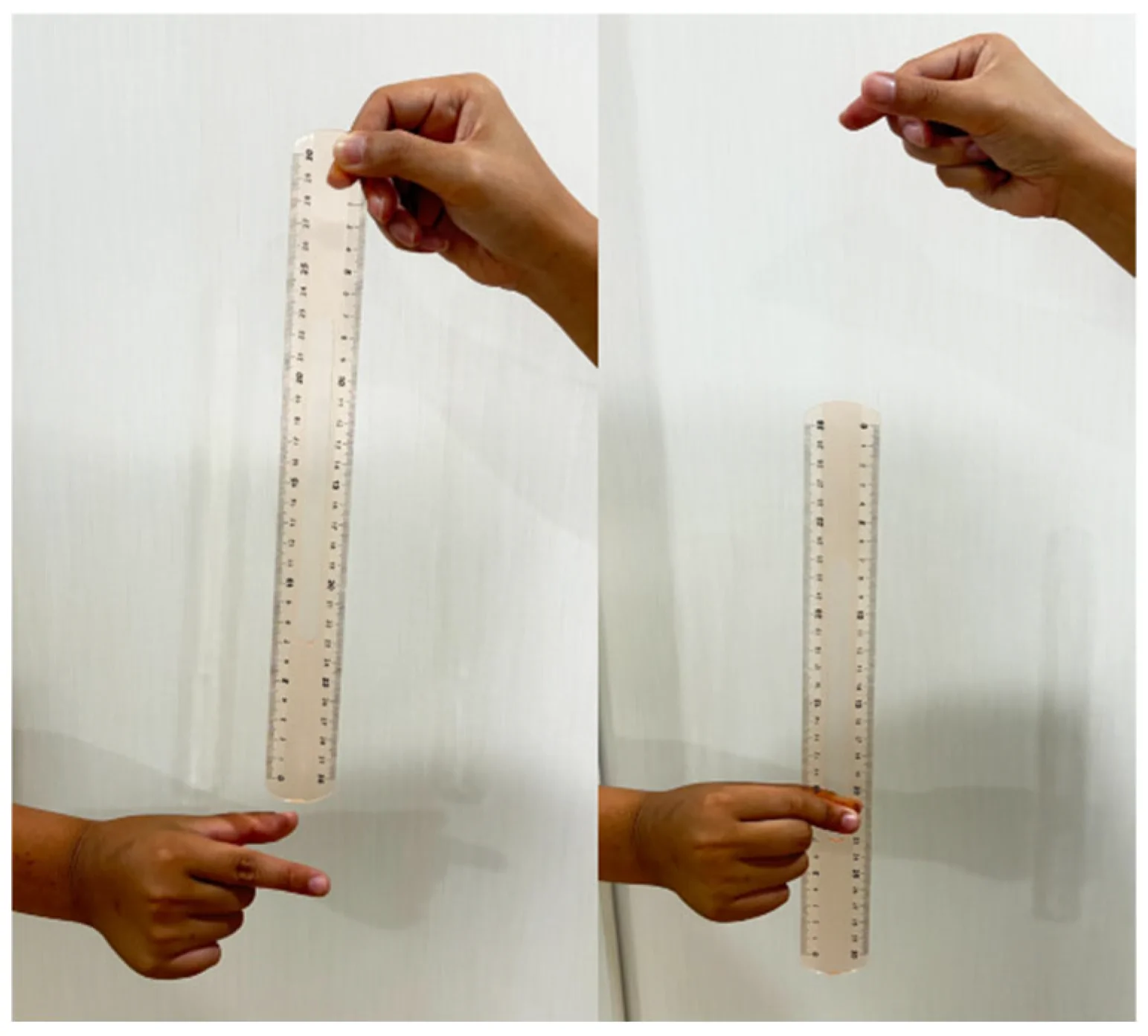
Ruler drop test setup used to assess simple visuomotor reaction speed.

For the RDT, participants stood upright and placed the thumb and index finger of their dominant hand slightly apart, without touching the ruler. An examiner held a vertically oriented 30 cm ruler above the participant’s hand, with the 0 cm mark aligned with the top of the participant’s thumb and index finger. Without prior warning, the examiner released the ruler, and participants attempted to catch it, as quickly as possible. One practice trial was completed before testing. The distance by which the ruler fell before it was caught was recorded in centimeters. Two trials were performed, and the mean distance was used as an index of simple visuomotor reaction speed (Figure 2).

### 2.5. Statistical Analysis

Descriptive statistics were calculated for all variables. Normality of continuous variables was assessed using the Shapiro–Wilk test; however, as some variables did not meet the assumption of normality, non-parametric methods were used for correlation analyses. Spearman’s rank correlation coefficients were calculated to examine the associations among age, TMT-A, RDT, and EHC. In addition, multiple linear regression analyses were conducted using EHC as the dependent variable. TMT-A completion time or RDT distance was included as an independent variable, and age and sex were included as covariates. Variance inflation factors (VIFs) were calculated to assess multicollinearity. For Model 1, linearity and homoscedasticity were evaluated by visual inspection of a scatterplot of standardized residuals against standardized predicted values. Approximate residual normality was evaluated using a histogram of the standardized residuals and a normal P–P plot. The diagnostic plots are provided in Supplementary Figure S1. Statistical significance was set at *p* < 0.05 (two-tailed).

Given the modest sample size (*n* = 27), bootstrap resampling was performed using SPSS to characterize sampling uncertainty around the regression coefficient estimates and their confidence intervals (CIs). Bias-corrected and accelerated (BCa) 95% CIs were calculated based on 2000 bootstrap samples. Ordinary least squares (OLS) estimates are reported as the primary results together with the corresponding bootstrap confidence intervals. Bootstrap resampling was not considered to increase statistical power, improve the stability of the coefficients, or address limited generalizability. Statistical analyses were performed using IBM SPSS Statistics (version 29; IBM Corp., Armonk, NY, USA).

## 3. Results

### 3.1. Participant Characteristics

The participant characteristics and task performance results are summarized in Table 1. The final sample consisted of 27 school-aged children, including 18 boys and 9 girls, with a mean age of 11.03 ± 1.58 years. For the visual–motor assessments, the mean RDT distance was 17.37 ± 3.60 cm, the mean TMT-A completion time was 33.37 ± 12.89 s, and the mean EHC completion time was 27.28 ± 2.09 s. Median (interquartile range [IQR]) values were 17.0 (5.0) cm for the RDT, 31.0 (10.0) s for TMT-A, and 26.92 (2.96) s for EHC. The corresponding ranges were 12–25 cm, 17–78 s, and 23.43–32.07 s, respectively.

### 3.2. Correlation Analysis

Shapiro–Wilk tests indicated that TMT-A completion time deviated significantly from normality (*W* = 0.841, *p* < 0.001), whereas RDT (*W* = 0.951, *p* = 0.233) and EHC (*W* = 0.978, *p* = 0.824) did not. Therefore, Spearman’s rank correlations were used for the correlation analyses.

Spearman’s rank correlations showed that age was significantly and negatively correlated with the RDT (*r* = −0.419, *p* < 0.05), TMT (*r* = −0.618, *p* < 0.01), and EHC (*r* = −0.643, *p* < 0.01). The RDT was positively correlated with TMT (*r* = 0.394, *p* < 0.05) and EHC (*r* = 0.397, *p* < 0.05). TMT and EHC were positively correlated (*r* = 0.542, *p* < 0.01) (Table 2). Exact *p*-values and 95% CI for all Spearman correlations are provided in Supplementary Table S2.

### 3.3. Multiple Regression Analysis

Multiple linear regression models were constructed with EHC completion time (s) as the dependent variable and age and sex as covariates (Table 3). In Model 1 (Age + Sex + TMT), the overall model was significant (adjusted *R*^2^ = 0.483; *F*(3,23) = 9.085; *p* < 0.001). TMT (*β* = 0.452, *p* = 0.010) and age (*β* = −0.403, *p* = 0.017) were significant predictors, whereas sex was not (*p* = 0.605). Bootstrap BCa 95% CI (2000 resamples) supported the findings (TMT B = 0.073 [0.035, 0.134]; age B = −0.533 [−1.019, −0.169]). In Model 2 (Age + Sex + Ruler Drop), the overall model was significant (adjusted *R*^2^ = 0.337; *F*(3,23) = 5.412; *p* = 0.006), but the RDT was not a significant predictor (*β* = 0.183, *p* = 0.316); age remained significant (*β* = −0.508, *p* = 0.009) but sex was not (*p* = 0.284). Bootstrap results were consistent (Ruler Drop B = 0.104 [−0.136, 0.302]; age B = −0.671 [−1.070, −0.174]). In Model 3 (Age + Sex + TMT + Ruler Drop), the overall model was significant (adjusted *R*^2^ = 0.463; *F*(4,22) = 6.604; *p* = 0.001). TMT (*β* = 0.433, *p* = 0.019) and age (*β* = −0.381, *p* = 0.040) were significant, whereas the RDT (*β* = 0.067, *p* = 0.699) and sex (*p* = 0.619) were not. Bootstrap BCa 95% CI likewise supported the conclusions (TMT B = 0.070 [0.024, 0.109]; age B = −0.365 [−0.941, −0.151]; Ruler Drop B = 0.032 [−0.175, 0.477]; sex B = −0.329 [−1.919, 0.770]). To evaluate the incremental contribution of TMT-A beyond age and sex, a hierarchical regression analysis was additionally conducted. The baseline model, including age and sex, explained 38.7% of the variance in the EHC performance (*R*^2^ = 0.387, adjusted *R*^2^ = 0.336, *F*(2,24) = 7.575, *p* = 0.003). After the inclusion of TMT-A, the explained variance increased to 54.2% (*R*^2^ = 0.542, adjusted *R*^2^ = 0.483), representing a significant increase of 15.5% (Δ*R*^2^ = 0.155, *F* change = 7.807, *p* = 0.010).

Visual inspection of the Model 1 diagnostic plots showed no clear curvilinear pattern or pronounced funnel-shaped dispersion in the scatterplot of standardized residuals against standardized predicted values. The residual histogram was almost symmetric and unimodal, and the points in the normal P–P plot generally followed the diagonal line. Thus, no evident major violations of linearity, homoscedasticity, or approximate residual normality were observed; however, these visual assessments were interpreted cautiously because of the small sample size (Supplementary Figure S1).

Multicollinearity diagnostics indicated no evidence of problematic collinearity among predictors, with VIF values ranging from 1.088 to 1.312. Participant 9 was identified as a potentially influential observation (Cook’s distance = 0.650, leverage = 0.557, studentized residual = 1.33, and studentized deleted residual = 1.36). A sensitivity analysis was therefore conducted by repeating Model 1 after excluding this observation. The overall model remained significant (*n* = 26; *R*^2^ = 0.466, adjusted *R*^2^ = 0.393, *F*(3,22) = 6.393, *p* = 0.003); however, the TMT-A coefficient was attenuated and was no longer statistically significant (*B* = 0.035, *SE* = 0.038, *β* = 0.176, 95% CI [−0.044, 0.114], *p* = 0.367). The incremental variance explained by TMT-A beyond age and sex decreased from 15.5% in the full sample (Δ*R*^2^ = 0.155, *p* = 0.010) to 2.1% after exclusion (Δ*R*^2^ = 0.021, *p* = 0.367). Bootstrap analysis yielded the same conclusion (bootstrap *p* = 0.298; 95% CI [−0.037, 0.115]). Thus, the adjusted association between TMT-A and EHC completion time was sensitive to this high-leverage observation.

Compared with Model 1, the inclusion of the RDT in Model 3 did not improve the model performance, as reflected by a lower adjusted *R*^2^ value (0.463 vs. 0.483). Furthermore, the RDT was not a significant predictor in Model 3 (*β* = 0.067, *p* = 0.699). These findings suggest that the RDT did not show an independent association with EHC after adjustment for age, sex, and TMT-A performance in the present sample.

## 4. Discussion

In this study, the relationships among visual search, simple visuomotor reaction speed, and EHC were examined in children aged 9–14 years. Both TMT-A performance and RDT scores correlated with EHC. In the full-sample multiple regression analysis, TMT-A, but not the RDT, was associated with EHC completion time after adjustment for age and sex. However, the TMT-A coefficient was substantially attenuated and became nonsignificant after exclusion of a high-leverage observation. Therefore, the adjusted association was not robust and should be interpreted as an exploratory finding rather than evidence of a stable independent relationship. Because the predictive contributions of TMT-A and the RDT were not formally compared and the measures differed in scale and reliability, the present results should not be interpreted as evidence that visual search or attention processes contribute more strongly to EHC than simple reaction speed.

Visual search and attention have been reported to improve with age, in late childhood and adolescence [7,17,18,19,20]. The findings of the present study were consistent with this developmental trend. In addition, longitudinal research demonstrated that EHC improves consistently from 8 to 16 years of age, with remarkable gains between 8 and 10 years, followed by a more gradual improvement thereafter [5].

The adjusted association between TMT-A and EHC may reflect shared task demands, including visual scanning, sequencing, processing speed, sustained attention, and graphomotor or upper-limb motor output. Because these cognitive and motor processes were not directly measured, this interpretation should be regarded as a hypothesis rather than evidence of a specific mechanism. Consistent with this interpretation, visual search and selective attention have been reported to mature from childhood to adolescence and improvements in cognitive processing capacity influence performance of visuomotor tasks [21,22,23,24].

Although TMT-A was associated with EHC completion time in the full-sample analysis, the RDT was not independently associated with EHC completion time after adjustment for age and sex. This difference should not be interpreted as evidence for EHC necessarily relying more strongly on the multidimensional demands involved in TMT-A than on simple reaction speed. The nonsignificant RDT coefficient may have reflected limited statistical power, measurement error arising from the manual procedure, or other task- and sample-specific characteristics.

One hypothesis is that the demands shared between TMT-A and the touchscreen task, such as visual scanning, processing speed, sequential responding, and upper-limb motor output, contributed to the association observed in the full sample. However, these mechanisms were not directly measured. Moreover, the sensitivity analysis showed that the TMT-A association was attenuated and no longer statistically significant after exclusion of a high-leverage observation. Accordingly, the differential findings for TMT-A and the RDT should be regarded as preliminary and hypothesis-generating rather than as evidence of distinct underlying mechanisms.

In addition, cognitive functions, such as visual search ability, selective attention, and visuospatial processing, improve rapidly from late childhood to adolescence (9–14 years) [5,18,19,20,21,22,23]. Therefore, the strong association between age and EHC, observed in this study, might reflect the developmental changes. In particular, a more efficient visual search and maturation of attentional control is closely related to improvements in the ability of EHC to accurately detect stimuli and transform them into appropriate motor responses. However, because this was a cross-sectional study, the age-related differences should not be interpreted as evidence of within-child developmental change. Cohort differences and selection effects cannot be excluded.

The absence of a significant sex effect should also be interpreted cautiously. The present sample included only nine girls and eighteen boys, which resulted in an imbalanced sex distribution. Therefore, the nonsignificant regression coefficient for sex should not be interpreted as evidence that sex is unrelated to EHC performance. Rather, the limited and unequal sample size may have reduced the precision of the estimated sex effect and the ability to detect potential sex-related differences.

The practical interpretation of the regression coefficient should also be considered. The practical significance of the observed association remains uncertain because no established clinical or functional thresholds for EHC performance are currently available. The present findings should, therefore, be interpreted as evidence of an association rather than evidence of clinical or functional relevance.

As a paper-based task, TMT-A may warrant future evaluation for potential use in school and community settings. However, feasibility, cost, acceptability, administration time, examiner training requirements, or implementation in these settings were not assessed in the present study. Therefore, its practical applicability is proposed rather than demonstrated. Several established visuomotor assessment tools, such as the Beery Visual–Motor Integration test, Movement Assessment Battery for Children, Purdue Pegboard Test, and Grooved Pegboard Test, provide valuable information regarding visual–motor integration, manual dexterity, and motor coordination [8,25,26,27]. These tests were not directly compared with TMT-A in the present study. Further research is needed to evaluate the feasibility and relative utility of these approaches in school and community settings.

Taken together, TMT-A performance was associated with EHC performance in the full sample after adjustment for age and sex, but this association was sensitive to exclusion of a high-leverage observation. Accordingly, this study was exploratory and the results presented here should not be interpreted as evidence of TMT-A functioning as a screening tool. Larger independent studies are needed to assess the stability of the association and to evaluate diagnostic accuracy, classification performance, and practical utility.

### Limitations

This study had several limitations. First, the sample size was small (*n* = 27), which limited statistical power and generalizability. Second, the age range was restricted to 9–14 years, and the findings may not apply to other age groups. Third, the cross-sectional design did not allow inference of developmental changes or causal relationships. Fourth, the RDT was manually recorded, which may have introduced measurement error. Additionally, the data were collected from a specific regional program, limiting external validity. Finally, only TMT-A was used as a visual search measure, and comparisons with other cognitive tasks were not performed. Future studies should include larger and more diverse samples, adopt longitudinal designs, and incorporate multiple visual tasks. Furthermore, diagnostic accuracy analyses, including sensitivity, specificity, classification thresholds, and external validation, were not performed. The present findings should therefore not be interpreted as evidence supporting the use of TMT-A as a screening tool for EHC.

A further limitation concerns model stability. One participant had high leverage and a Cook’s distance above the sample-sensitive threshold of 4/*n*. Excluding this observation reduced the TMT-A coefficient and rendered its incremental contribution nonsignificant. The adjusted association observed for the full sample may therefore depend materially on this individual observation, underscoring the uncertainty of the regression estimate for this small sample.

The outcomes of both TMT-A and touchscreen assessments were timed upper-limb visuomotor tasks. Their observed association may therefore reflect general processing speed, overall task-completion speed, graphomotor or upper-limb motor demands, or other shared method variance rather than correspondence with the broader EHC construct. Accordingly, the present findings should not be interpreted as evidence that TMT-A measures EHC as a broad construct.

The unequal sex distribution (18 boys and 9 girls) may have limited the ability to detect potential sex-related differences in EHC performance. Another limitation is that prior touchscreen experience, familiarity with digital devices, educational background, and physical activity or sports experience were not assessed. These factors may influence performance on both the touchscreen-based EHC task and the TMT-A, but they were not measured because this exploratory study was designed to minimize participant burden in a community setting. Therefore, these variables could not be included as covariates and should be considered potential unmeasured confounders. In addition, information regarding corrected visual acuity, color vision, handedness, neurological conditions, developmental disorders, learning disabilities, ADHD, and medication use was not collected systematically. Therefore, these factors may also have influenced performance on the TMT-A, RDT, and touchscreen-based EHC task.

Furthermore, objective assessments of visual acuity, refractive error, binocular vision status, and ocular health were not performed. Participants were included based on self-reported visual function sufficient for daily activities. Therefore, unrecognized visual abnormalities may have influenced performance on both the TMT-A and EHC tasks.

The touchscreen-based EHC outcome should also be interpreted cautiously. Although the V-training system has been used in previous studies to assess visuomotor and visual functions, including EHC and spatial awareness, its external validity and test–retest reliability as a criterion measure of EHC in school-aged children have not been fully established. Therefore, the EHC outcome should be regarded as task-specific touchscreen-based visuomotor performance rather than a validated gold-standard measure of EHC.

Participant-specific randomization of target locations may have introduced variations in task difficulty. Although randomization reduced target predictability and the potential for sequence-related learning effects, differences in target sequence and spatial distribution may have affected visual-search demands, movement distance, and completion time across participants. The equivalence of task difficulty across randomized target layouts was not formally evaluated, and this variability may have contributed to measurement error.

The present study did not include trial-level reliability analyses for the repeated RDT trials, which may have introduced measurement uncertainty. Accuracy-related measures and error data were not recorded and were therefore not analyzed.

Developmental effects may also not have been fully controlled by including age as a single linear covariate because the sample covered a period of rapid and potentially nonlinear development. Although TMT-A is practically accessible, feasibility, administration time, cost, examiner training, or acceptability in school or community settings were not directly evaluated. In addition, the validity of the specific Japanese-language TMT-A version used in this study for school-aged children was not formally evaluated.

## 5. Conclusions

In the full sample, TMT-A performance was associated with EHC completion time after adjustment for age and sex; however, this association was not robust to exclusion of a high-leverage observation. The study findings should therefore be considered exploratory and require confirmation in a larger independent sample before the diagnostic accuracy, reliability, and practical utility of TMT-A for screening purposes can be determined.

## Figures and Tables

**Table 1:** Participant characteristics and task performance (*n* = 27).

Variable	Mean ± SD or *n* (%)
Age (years)	11.03 ± 1.58 (Range: 9–14)
Sex	Male: 18 (66.7%), Female: 9 (33.3%)
Visual–motor test	
Ruler drop test (cm)	17.37 ± 3.60
Trail making test (s)	33.37 ± 12.89
Eye–hand coordination (s)	27.28 ± 2.09

Note: Values are means ± SD or *n* (%). The ruler drop was measured in cm, and TMT and EHC were measured in s. Age distribution was as follows: 9 years, 4 (14.8%); 10 years, 8 (29.6%); 11 years, 7 (25.9%); 12 years, 2 (7.4%); 13 years, 3 (11.1%); 14 years, 3 (11.1%).

**Table 2:** Spearman’s rank correlations among study variables (*n* = 27).

Variable	Age	Ruler Drop Test (cm)	TMT (s)	EHC (s)
Age	—	−0.419 *	−0.618 **	−0.643 **
Ruler drop test (cm)	−0.419 *	—	0.394 *	0.397 *
TMT (s)	−0.618 **	0.394 *	—	0.542 **
EHC (s)	−0.643 **	0.397 *	0.542 **	—

Note. Spearman’s rank correlation coefficients are presented. * *p* < 0.05, ** *p* < 0.01 (two-tailed). Lower scores on the TMT/EHC indicate faster performance.

**Table 3:** Multiple regression analyses predicting EHC completion time (s); OLS estimates with bootstrapped BCa 95% confidence intervals (2000 resamples).

**Model 1 (Age + Sex + TMT)** *Model fit:* Adjusted *R*^2^ = 0.483; *F*(3,23) = 9.085; ***p*** **< 0.001**							
**Predictor**	**B (OLS)**	**SE B**	***β***		***t***	***p***	**BCa 95% CI for B**
TMT (s)	0.073	0.026	0.452		2.794	0.010	[0.035, 0.134]
Age (years)	−0.533	0.206	−0.403		−2.585	0.017	[−1.019, −0.169]
Sex (ref. = Female)	−0.339	0.647	−0.077		−0.524	0.605	[−1.914, 0.770]
**Model 2 (Age + Sex + Ruler Drop)**—*Model fit:* Adjusted *R*^2^ = 0.337; *F*(3,23) = 5.412; ***p*** **= 0.006**							
**Predictor**	**B (OLS)**	**SE B**	***β***		***t***	***p***	**BCa 95% CI for B**
Age (years)	−0.671	0.235	−0.508		−2.853	0.009	[−1.070, −0.174]
Ruler Drop (cm)	0.104	0.102	0.183		1.026	0.316	[−0.136, 0.302]
Sex (ref. = Female)	−0.768	0.699	−0.176		−1.098	0.284	[−2.201, 0.736]
**Model 3 (Age + Sex + TMT + Ruler Drop)** *Model fit:* Adjusted *R*^2^ = 0.463; *F*(4,22) = 6.604; ***p*** **= 0.001**							
**Predictor**	**B (OLS)**	**SE B**	***β***		***t***	***p***	**BCa 95% CI for B**
TMT (s)	0.070	0.028	0.433		2.589	0.019	[0.024, 0.109]
Ruler Drop (cm)	0.032	0.088	0.067		0.390	0.699	[−0.175, 0.477]
Age (years)	−0.365	0.169	−0.381		−2.161	0.040	[−0.941, −0.151]
Sex (ref. = Female)	−0.329	0.653	−0.076		−0.505	0.619	[−1.919, 0.770]
**Additional model diagnostics**							
**Item**				**Value**			
Δ*R*^2^ (TMT beyond age and sex)				0.155			
*F* change				7.807			
*p*				0.010			
VIF range				1.088–1.312			
Standardized residuals				−1.569 to 1.713			
Maximum Cook’s distance				0.650			

Notes. Sex was coded as 0 for females (reference) and 1 for males. EHC = Eye–Hand Coordination; TMT = Trail Making Test; OLS = ordinary least squares; BCa CI = bias-corrected and accelerated confidence interval. Ruler Drop was measured in cm. BCa 95% confidence intervals were based on 2000 bootstrap resamples. Hierarchical regression showed that TMT-A increased the explained variance beyond age and sex in the full sample (Δ*R*^2^ = 0.155, *F* change = 7.807, *p* = 0.010). Participant 9 had the maximum Cook’s distance (0.650), leverage of 0.557, studentized residual of 1.33, and studentized deleted residual of 1.36. After exclusion of participant 9, the increment in TMT-A was reduced and was not statistically significant (Δ*R*^2^ = 0.021, *p* = 0.367).

## Data Availability

The data presented in this study are available upon reasonable request from the corresponding author.

## Supplementary Materials

The following supporting information can be downloaded at https://www.mdpi.com/article/10.3390/life16091421/s1: Table S1. Participant-level demographic characteristics; Table S2. Exact *p*-values and 95% confidence intervals for Spearman correlations; Figure S1. Regression diagnostic plots for Model 1 (full sample, *n* = 27): (A) histogram of standardized residuals, and (B) normal P–P plot of standardized residuals, (C) standardized residuals versus standardized predicted values.

## Abbreviations

The following abbreviations are used in this manuscript:

EHC	Eye–Hand Coordination
TMT	Trail Making Test
RDT	Ruler Drop Test
