# Supplementary material for: Association Between Visual Search Performance and Eye–Hand Coordination in School-Aged Children
Source: Life (Basel). 2026 Aug 27;16(9):1421. doi: 10.3390/life16091421 (PMC13608825; doi:10.3390/life16091421)
Supplement: Supplementary file 1 [file life-16-01421-s001.zip › Supplementary Figure S1.pdf]

Supplementary Figure S1. Regression diagnostic plots for Model 1 (full sample, n = 27).

(A) Histogram of standardized residuals.

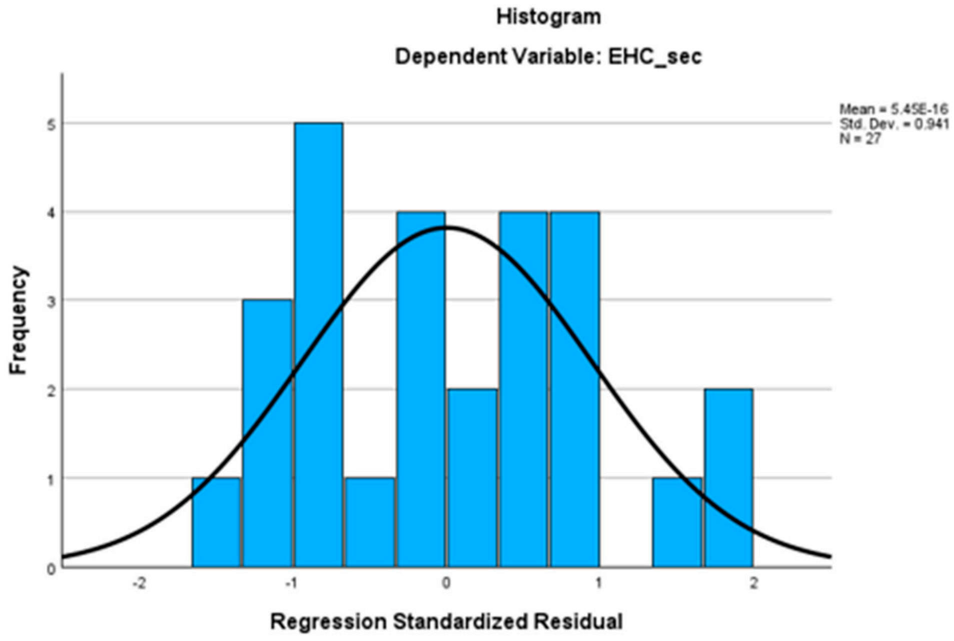

(B) Normal P–P plot of standardized residuals.

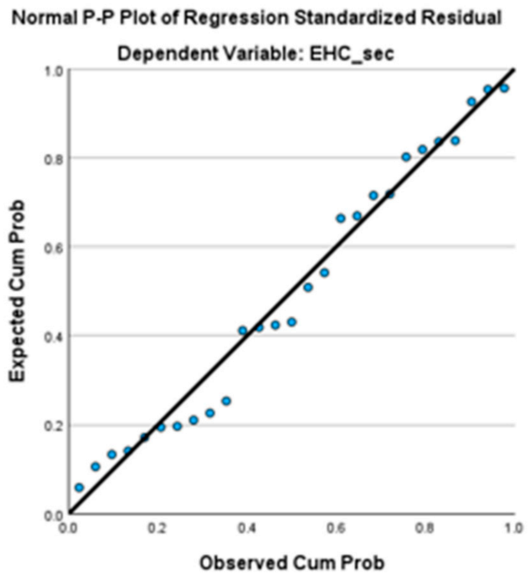

(C) Standardized residuals versus standardized predicted values.

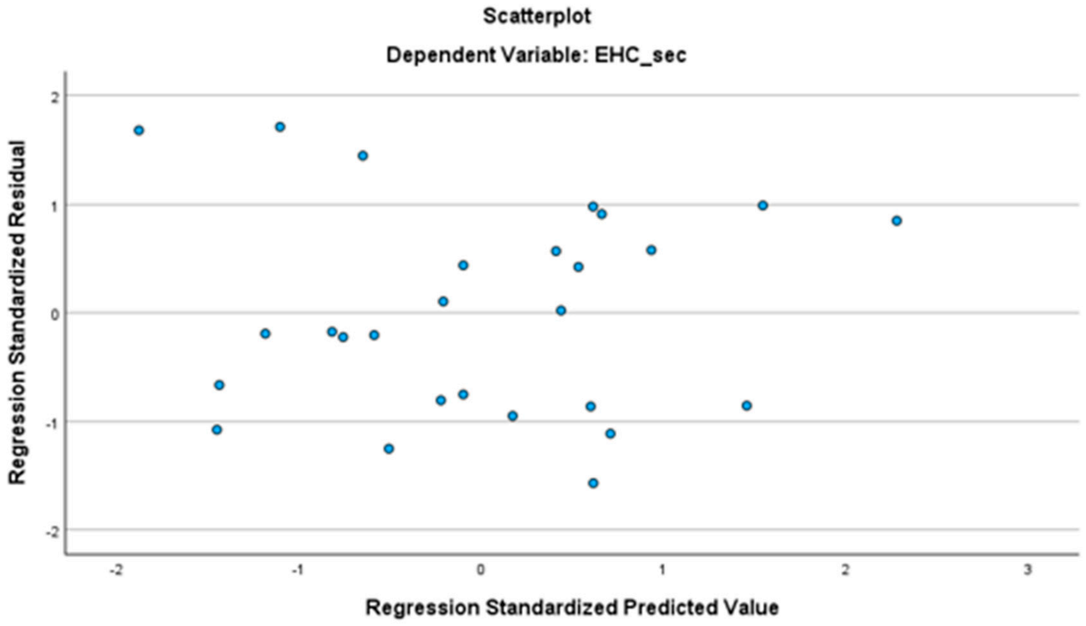
Scatterplot
Dependent Variable: EHC_sec
Regression Standardized Residual
2
1
0
-1
-2
-2
-1
0
1
2
3
Regression Standardized Predicted Value
